# Activity Based High-Throughput Screening for Novel O-GlcNAc Transferase Substrates Using a Dynamic Peptide Microarray

**DOI:** 10.1371/journal.pone.0151085

**Published:** 2016-03-09

**Authors:** Jie Shi, Suhela Sharif, Rob Ruijtenbeek, Roland J. Pieters

**Affiliations:** 1 Department of Medicinal Chemistry and Chemical Biology, Utrecht University, Utrecht, The Netherlands; 2 PamGene International BV, ‘s-Hertogenbosch, The Netherlands; Bangor University, UNITED KINGDOM

## Abstract

O-GlcNAcylation is a reversible and dynamic protein post-translational modification in mammalian cells. The O-GlcNAc cycle is catalyzed by O-GlcNAc transferase (OGT) and O-GlcNAcase (OGA). O-GlcNAcylation plays important role in many vital cellular events including transcription, cell cycle regulation, stress response and protein degradation, and altered O-GlcNAcylation has long been implicated in cancer, diabetes and neurodegenerative diseases. Recently, numerous approaches have been developed to identify OGT substrates and study their function, but there is still a strong demand for highly efficient techniques. Here we demonstrated the utility of the peptide microarray approach to discover novel OGT substrates and study its specificity. Interestingly, the protein RBL-2, which is a key regulator of entry into cell division and may function as a tumor suppressor, was identified as a substrate for three isoforms of OGT. Using peptide Ala scanning, we found Ser 420 is one possible O-GlcNAc site in RBL-2. Moreover, substitution of Ser 420, on its own, inhibited OGT activity, raising the possibility of mechanism-based development for selective OGT inhibitors. This approach will prove useful for both discovery of novel OGT substrates and studying OGT specificity.

## Introduction

While carbohydrates are typically prominently displayed on cellular surfaces, the display of N-acetyl glucosamine (GlcNAc) residues on serine and threonine residues is also a very common and important occurrence inside the cell. Not unlike the role of phosphate groups, GlcNAc groups play important roles in various signaling cascades that control important cellular processes. Such processes include transcription, cell cycle regulation, stress response and protein degradation. Furthermore there is a significant connection between an unbalance of GlcNAcylation and diseases such as cancer, diabetes and neurodegenerative disease. Improved understanding of GlcNAcylation has the potential to lead to new therapeutics [1, 2] and diagnostics[3].

Surprisingly only a limited number of enzymes are involved in the dynamic attachment and removal of GlcNAc moieties. For the attachment it is the O-GlcNAc transferase (OGT) and for the removal O-GlcNAcase (OGA). OGT exists in three isoforms: nc-OGT, m-OGT and s-OGT which differ in the number of tetratricopeptide repeats are attached to the identical catalytic domain. The nucleocyctoplasmic (nc) isoform contains 12.5 repeats, the mitochondrial (m) isoform 9.5 repeats and the short (s) isoform contains only 2.5 repeats. While there are hundreds of kinases that are more or less selective, such selectivity in the O-GlcNAcylation process is likely caused by additional proteins that transiently associate to the OGT isoforms and possibly the inherent substrate specificity.

O-GlcNAc levels correlate with the amount of available UDP-GlcNAc which is linked to the cellular glucose levels[4]. Elevated O-GlcNAcylation (or hyper O-GlcNAcylation) was shown to occur in various types of cancer[5]. In line with this, reducing the O-GlcNAcylation level blocks tumor growth[6]. Due to the different metabolism of cancer cells, relying more on glycolysis instead of oxidative phosphorylation, more glucose is needed which also leads to elevated O-GlcNAcylation. Relevant to diabetes is the fact that high glucose levels increase the GlcNAcylation of proteins within the insulin signaling pathway which contributes to insulin resistance[5]. With respect to Alzheimer’s disease, several proteins involved in the disease, are normally GlcNAcylated and less so in case of the disease as a consequence of reduced glucose uptake[7, 8].

The interplay between phosphorylation and GlcNAcylation is increasingly being recognized as an important yet complex phenomenon in cell signaling. Many sites of proteins that become phosphorylated can also become GlcNAcylated[9, 10], and also kinases are frequently GlcNAcylated[11]. The phosphorylation can be at the same location as the GlcNAcylation, or also at nearby serines and threonines and even tyrosine phosphorylation is known to interplay with O-GlcNAc modification. For the case of the tau kinase, of relevance in Alzheimer’s disease, it was recently shown that a different conformational change is induced by either phosphorylation or GlcNAcylation[12]. While phosphorylation is associated with protein misfolding and aggregation, O-GlcNAcylation stabilizes the soluble form of tau.

There are several ways to study GlcNAcylation[13]. One way is the use of metabolic chemical reporters, which in this case are azide derivatized GlcNAc molecules that are being incorporated into biosynthetic pathways and are transferred by OGT to its substrate proteins. Lysed cells are subsequently treated with fluorescent labels, which are attached to the GlcNAc by CuAAC and analyzed on a gel by fluorescence. Typically, azide derivatives are being used with the azido group placed at either the N-acetyl group (as in UDP-GlcNAz) or at the 6-position[14]. UDP-GalNAz was also used on His-tagged substrate proteins on Ni-NTA covered microplates, where a ligation of the GlcNAz’s azido function led to clearly visible fluorescence[15].

Considering the importance of GlcNAcylation and its interplay or crosstalk with phosphorylation, it is of importance to know which proteins and which protein sequences are being modified. By studying protein X-ray structures it was found that that GlcNAcylation sites are relatively often found in protein sequences with random coil conformations and rarely in areas with secondary structures[16]. Since this suggested that primary structure would be the deciding factor, a consensus sequence was sought. Using a database search and GlcNAcylation experiments by s-OGT of a series of peptides and protein mutants, preferred sequences were identified. The most frequent amino acid residues found were Pro/Ala (at positions -2 relative to the GlcNAcylated serine or threonine), and Val/Ala/Thr (at -1) and Ala/Ser/Pro/Thr/Gly (at +2) [16, 17].

Mass spectrometry is the most commonly used method and its strengths are well known. Nevertheless there are also weaknesses for identifying GlcNAcylation such as the detection of false positives or missing low abundance proteins. Furthermore site mapping of GlcNAcylated positions is difficult due to the relatively weak link between sugar and protein and the low stoichiometry. In many cases enrichment methods were used e.g. by GlcNAc specific lectins such as WGA [18]. Due to the enrichment step large amounts of protein are needed for a mass spectrometric analysis. In another example the GlcNAc residues in a proteolytically digested lysate are enzymatically extended with a GalNAz group, whose azido function is subsequently linked to biotin, thus allowing enrichment [19]. MRM (multiple Reaction Monitoring)-MS was recently shown to be a promising method for the qualitative and quantitative assessments of O-GlcNAcylated peptides issued from native proteins[20].

Recently a protein microarray method was described that led to the identification of about a dozen validated new protein substrates of nc-OGT and m-OGT[21]. A commercial array of ca 8000 human proteins was exposed to OGT/UDP-GlcNAc followed by antibody detection of GlcNAcylation. Preexisting GlcNAcylated proteins were separately detected and subtracted. Validation of identified hits was achieved by using UDB-GlcNAz, which allowed click functionalization of attached GlcNAz that was visualized on a gel. Clearly, the use of a protein array is useful, although the actual GlcNAcylation location is not readily identified, even though this is of importance e.g. for detailed studies where the interplay with kinases is studied. In the kinase field the use of immobilized peptide sequences derived from knowledge of protein phosphorylation sites has proven to be highly valuable for the study of kinase activities[22]. This activity can be limited to a single kinase but also a tissue lysate can be used and the kinase activity profile has been shown to be of relevance for cancer target discovery[23], diagnosis, and for the prediction of the efficacy of kinase inhibiting drugs for a particular patient[24]. We here report on an application of the peptide array approach to identify GlcNAcylation activity and study its specificity. Interestingly, the protein RBL-2 was identified as an OGT substrate for all three isoforms. This protein is a key regulator of entry into cell division and thus has relevance for cancer. The recent disclosure of a similar discovery [25]prompted us to disclose our new method.

## Materials and Methods

### Materials

Uridine 5′-diphospho-N-acetylglucosamine sodium salt (U4375) and isopropyl 1-thio-β-D-galactopyranoside was purchased from sigma Aldrich (Zwijn-drecht, The Netherlands). The mouse monoclonal anti-Anti-O-Linked N-Acetylglucosamine antibody [RL2] (ab2739) was from Abcam (London, England). FITC conjugated secondary antibody was purchased from Thermo scientific (Bleiswijk, Netherland). A known OGT inhibitor 3-(2-adamantanylethyl)-2-[(4-chlorophenyl) azamethylene]-4-oxo-1,3-thiazaperhyd roine-6-carboxylic acid was obtained from TimTec (Newark, USA). All PamChip 4 microarray chips were provided by PamGene (Pamgene international, The Netherlands).

### Plasmid constructs and recombinant OGT isoforms overexpression

Plasmids encoding s-OGT, m-OGT, and nc-OGT are generous gifts from John A. Hanover (National Institute of Health, USA). OGT overexpression was carried out as previously described[26]. All the plasmids were transformed into E.coli BL21 (DE3) competent cells to produce a cell lysate containing OGT for use or for further purification. Briefly, inoculated a single colony into 50 ml of LB broth media containing 50 μg/ml ampicillin and cells were grown overnight at 37°C with vigorous shaking. 50 mL of the start culture was added into 1L LB broth media containing 50 μg/mL ampicillin and it was incubated with shaking at 200 rpm at 37°C until A600 reached 0.4–0.6. After the culture was cooled to room temperature, 0.5 mM IPTG was added into the culture to induce protein expression. After 16 hours, the cells were collected by centrifugation for 10 min at 4000 RPM at 4°C. The pellets were lysed in lysis buffer containing 20 mM Tris-HCl, pH 7.5, 1 mg/mL lysome, 0.1% triton x-100 and complete mini EDTA-free protease inhibitor cocktail on ice for 10 min and the lysate was sonicated until the DNA was completely sheared. After centrifugation for 10 min at 12000 rpm at 4°C, the supernatant was carefully transferred into a clean tube and was ready for the array assay or for further purification. Protein expression was determined by coomassie blue staining and western blotting using an anti-OGT antibody.

### Recombinant OGT isoform purification

One step OGT purification was performed using His60 Ni Gravity Flow Columns (Clontech) according to the manufacturer’s instructions. Briefly, the nickel column was equilibrated with a buffer containing 50 mM sodium phosphate, 300 sodium chloride, 20 mM imidazole, and pH 7.4, followed by incubation with cell lysate containing His tag OGT for 1h at 4°C. After washing several times using buffer containing 50 mM sodium phosphate, 300 sodium chloride, 40 mM imidazole, and pH 7.4, the recombinant OGT was eluted using elution buffer containing 50 mM sodium phosphate, 300 mM sodium chloride, 300 mM imidazole, and pH 7.4. The eluted fraction was concentrated through a 100,000 Da molecular weight cut off Amicon unit by centrifugation 10 min at 5000 RPM at 4°C.

### Microarray analysis of peptide O-GlcNAcylation

Peptide O-GlcNAcylation was assessed using PamChip 4 microarray chips with a PamStation 12 instrument (Pamgene international, The Netherlands). The OGT enzyme reaction was prepared essentially as described[26] with minor modifications. Briefly, before addition of the enzyme reaction, the microarray was blocked with blocking buffer (1% BSA in TBS). After 30 cycles (2 cycles/min) blocking, the blocking buffer was aspirated and the microarray was incubated with 40 μL of the enzyme reaction containing OGT (lysate or purified), OGT reaction buffer (50 mM Tris—HCl, pH 7.5;1 mM DTT;12.5 mM MgCl2), a 1:1 pre-incubated mixture of anti-O-GlcNAc and FITC conjugated secondary antibody, and the sugar donor UDP-GlcNAc or water as a control for 480 cycles (2 cycles/min) at 30°C. The microarray was run in parallel by pumping the reaction up and down through the porous membrane at every cycle and a tiff image was obtained at every 20 cycles by a CCD camera inside the PamStation. O-GlcNAcylation of each peptide was detected by a fluorescent signal which was produced by an FITC conjugated antibody bound to the O-GlcNAc moiety on the peptide.

Image analysis was performed using BioNavigator 6 software (Pamgene international, The Netherlands). Briefly, each image was quantified by automated array grid finding and subsequent quantification of signal and local background for each individual spot. In this work, the signal median-minus-background value was used as the quantitative parameter for the O-GlcNAcylation of the peptide.

### *In vitro* UDP-Glo assay

In this work OGT activity was also determined using UDP-Glo^™^ Glycosyltransferase Assay according to the manufacturer’s instructions. This assay evaluates O-GlcNAcylation through monitoring UDP formation in glycosyltransferase reactions by luminescence. Briefly, OGT reactions were carried out in 100 μL final volume containing 0.5 mM UDP-GlcNAc, 6 μg purified/60 μg lysate enzyme, 100 μM peptide in OGT reaction buffer (50 mM Tris—HCl, pH 7.5;1 mM DTT;12.5 mM MgCl_2_). Reactions were incubated at room temperature for 2h. At the end of that period, each reaction was transferred in triplicate into a 96-well white microplate and was mixed with a 1:1 ratio of UDP-Glo Detection Reagent. After incubation at room temperature for 1h, the luminescence was recorded with a Mithras LB940 Multimode microplate reader using Mikro Win 2000 software (Berthold Technology, Germany).

### Peptide synthesis and LC-MS anaylsis

Synthesis of all the peptides was achieved by following a standard Fmoc SPPS strategy on a Symphony Multiple Peptide Synthesizer and starting from rink amide resin. The following Fmoc amino acids were used: Fmoc-Cys(Trt)-OH, Fmoc-Gly-OH, Fmoc-Lys(Boc)-OH, Fmoc-Glu(Otbu)-OH, Fmoc-Asn(Trt)-OH, Fmoc-Ser(tBu)-OH, Fmoc-Thr(tBu)-OH, Fmoc-Ala-OH, Fmoc-Pro-OH, Fmoc-Val-OH. Deprotection was performed using 20% piperidine in DMF, and coupling was performed using 1:0.9:2 amino acid/ HBTU/DIPEA in DMF. After completion of the synthesis, the protected peptidyl resins were incubated with a 10 mL mixture of TFA (trifluoroacetic acid): H_2_O: triisopropylsilane (TIPS): 1, 2-ethanedithiol (EDT) (9:0.5:0.25:0.25, v/v/v/v) and allowed to stir for 2 h at room temperature under a nitrogen atmosphere. The cleaving mixture was filtered and the resin was washed with TFA (2 mL) and DCM (4 mL). The residue was precipitated by treatment of precooled diethyl ether and centrifugation. Two times washing and centrifugation yielded the crude products in a pellet. The precipitated peptides were dissolved in water, frozen, and lyophilized. All products were stored at -20°C. Crude peptide analysis was carried out by LC-MS.

## Results

### Identification by peptides microarrays of OGT targets from kinase substrates and co-regulators of nuclear receptors

**Fig 1:**
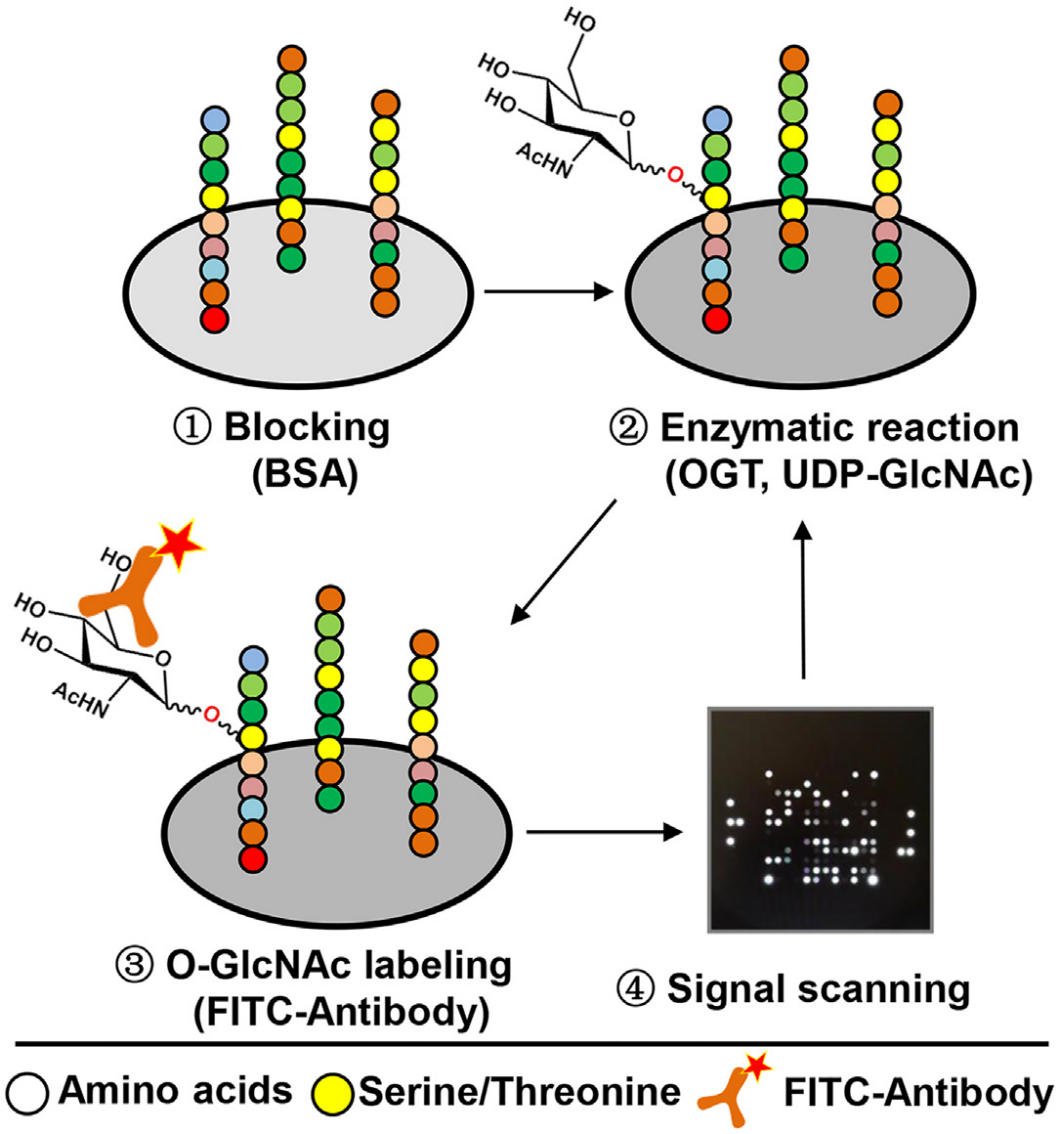
The schematic depiction of the peptide microarray process for discovering OGT substrates. The microarray was blocked with BSA, and followed by the addition of OGT (s-OGT, m-OGT, and nc-OGT), UDP-GlcNAc, and a FITC conjugated antibody. The enzymatic reaction was run by pumping the mixture up and down through the porous Al_2_O_3_ chip material. Images of the fluorescent signals were generated every 10 minutes during 4 hours (kinetic readout). Images were quantified using Bionavigator 6 software.

**Fig 2:**
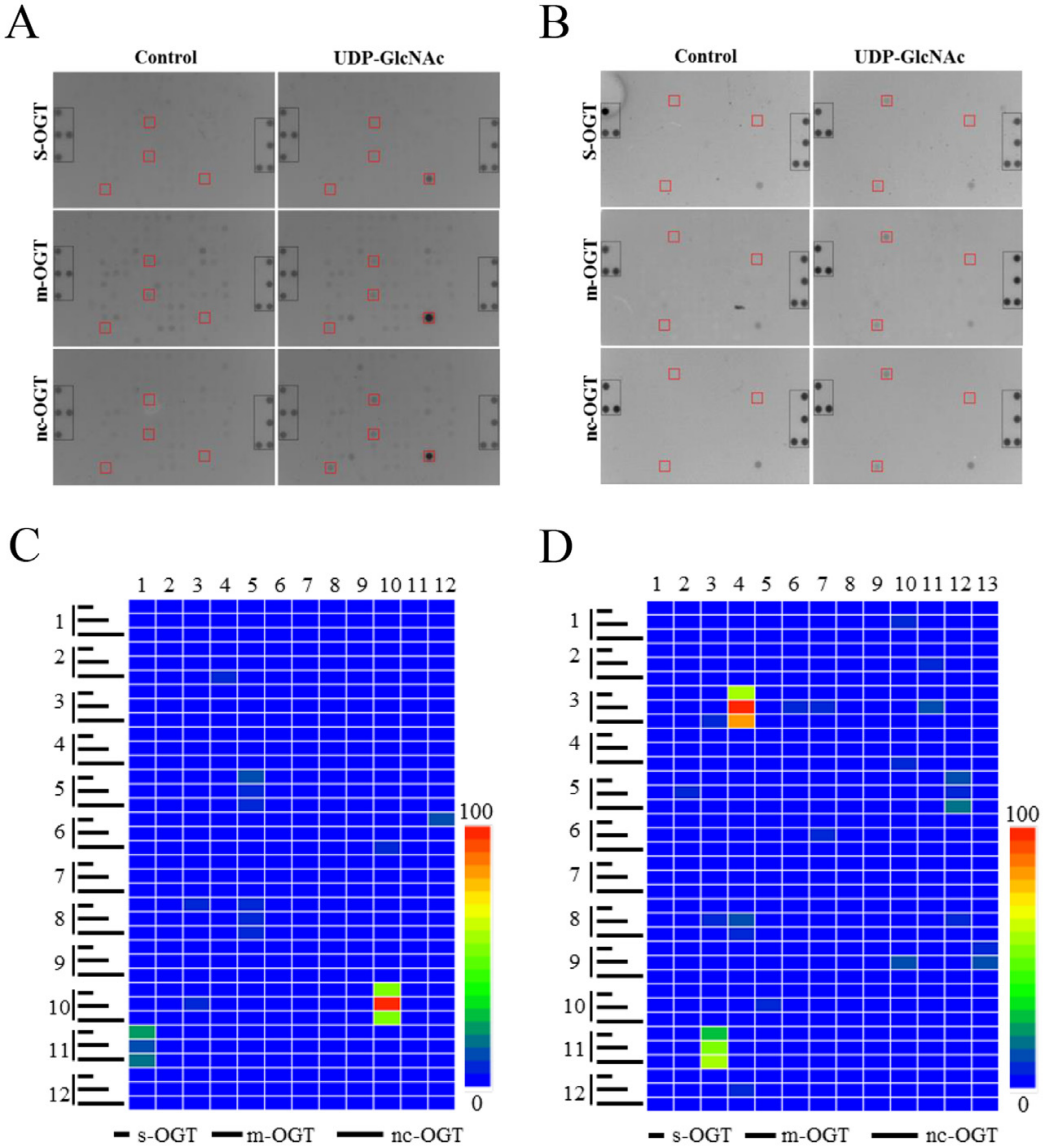
Identification of human OGT substrates from a kinase substrate and nuclear hormone receptor interaction peptide microarray. The assay was performed using bacterial lysates containing s-OGT (7 μg/μL), m-OGT (7 μg/μL), or nc-OGT (7 μg/μL), respectively, and in all cases in the presence of UDP-GlcNAc (1 mM). A parallel reaction without UDP-GlcNAc was used as a negative control. Representative images from the kinase substrate peptide microarray (A) and nuclear receptor interaction peptide microarray (B) are shown. Reference spot is highlighted in gray and peptide O-GlcNAcylation by all three isoforms of OGT is highlighted in red. O-GlcNAcylation of each peptide by the three isoforms OGT was quantified and corrected for non-specific signal by subtracting the signal generated without UDP-GlcNAc (from signal with UDP-GlcNAc). Representative heat maps are shown for O-GlcNAcylation of kinase substrate peptides (C) and nuclear receptor interaction peptides (D).

Dynamic peptide microarray (PamChip) technology has been successfully and widely used for kinase activities profiling in the last decade [27, 28]. In the present work, we describe, for the first time, the utility of this peptide microarray format for the identification of human OGT activity. Due to the fact that O-GlcNAcylation and phosphorylation may occur at the same or proximal amino acids on the same protein [9], a peptide microarray of 144 peptides derived from different kinase substrates (S1 Appendix) was selected for an initial test. As shown in Fig 1, upon blocking the microarray with 1% BSA (in TBS), an enzymatic reaction was initiated by adding a mixture of OGT reaction buffer, bacterial lysate containing recombinant OGT (s-OGT, m-OGT, nc-OGT), the sugar donor UDP-GlcNAc, and FITC conjugated O-GlcNAc antibody. O-GlcNAcylation of peptides in each individual spot was detected by a fluorescent signal produced by the FITC labelled antibody recognizing the O-GlcNAcylated amino acids of converted peptides. Kinetic activity was measured through scanning the signal at regular intervals. In this work, bacterially expressed recombinants of the three isoforms of OGT (s-OGT, m-OGT, nc-OGT) were used to catalyze the attachment of GlcNAc onto the target peptides, and we found that among these peptides one derived from GYS2, one derived from MYPC3, and two derived from RBL-2 were significantly O-GlcNAcylated by all three isoforms OGT (Fig 2A and Table 1). Similarly, a peptide microarray (S2 Appendix) containing 155 nuclear hormone receptor binding peptides derived from co-activators was tested and O-GlcNAcylation by three isoforms was observed on three peptides which were derived from NOCA6, BRD8, WIPI2, respectively (Fig 2B and Table 2).

**Table 1:** A list of OGT substrates identified on kinase substrate peptide microarray.

Location		Peptide	Sequence	OGT isoforms activity		
Row	Col			s	m	nc
2	4	BCKD_45_57 *	ERSKTVTSFYNQS	N	N	P
5	5	GYS2_1_13	MLRGRSLSVTSLG	P	P	P
6	10	KCNB1_489_501	KWTKRTLSETSSS	N	N	P
6	12	KPB1_1011_1023	QVEFRRLSISAES	P	N	N
8	3	MYBB_513_525	DNTPHTPTPFKNA	P	N	N
8	5	MYPC3_268_280	LSAFRRTSLAGGG	P	P	P
10	3	KIF2C_105_118_S106G	EGLRSRSTRMSTVS	N	P	N
10	10	RBL-2_410_422 *	KENSPCVTPVSTA	P	P	P
11	1	RBL-2_655_667*	GLGRSITSPTTLY	P	P	P

Signal greater than 5% of the highest signal was defined as positive (P), otherwise negative (N).

*peptides were shown to be O-GlcNAcylated recently[25].

**Table 2:** A list of OGT substrates identified on nuclear hormone receptor interaction peptide microarray.

Location		Peptide	Sequence	OGT isoforms activity		
Row	Col			s	m	nc
1	10	DHX30_241_262	QFPLPKNLLAKVIQIATSSSTA	N	P	N
2	11	NCOA2_866_888	SQSTFNNPRPGQLGRLLPNQNLP	N	P	N
3	3	NCOA3_MOUSE_1029_1051	HGSQNRPLLRNSLDDLLGPPSNA	N	N	P
3	4	NCOA6_1479_1501	LVSPAMREAPTSLSQLLDNSGAP	P	P	P
3	11	NRIP1_121_143_P124R	DSVRKGKQDSTLLASLLQSFSSR	N	P	N
4	10	MED1_591_614	HGEDFSKVSQNPILTSLLQITGNG	N	N	P
5	2	NR0B2_9_31_C9S/C11S	SPSQGAASRPAILYALLSSSLKA	N	P	N
5	12	BRD8_254_276	TVAASPAASGAPTLSRLLEAGPT	P	P	P
8	3	NRIP1_8_30	GSDVHQDSIVLTYLEGLLMHQAA	N	P	N
8	4	NRIP1_253_275_C263S	PATSPKPSVASSQLALLLSSEAH	N	P	P
9	13	PRGR_42_64_C64S	SDTLPEVSAIPISLDGLLFPRPS	P	P	N
10	5	LCOR_40_62	TTSPTAATTQNPVLSKLLMADQD	N	P	N
11	3	WIPI1_313_335_C318S	GQRNISTLSTIQKLPRLLVASSS	P	P	P

Signal greater than 5% of the highest signal was defined as positive, otherwise negative.

### RBL-2 is a possible target of OGT

**Fig 3:**
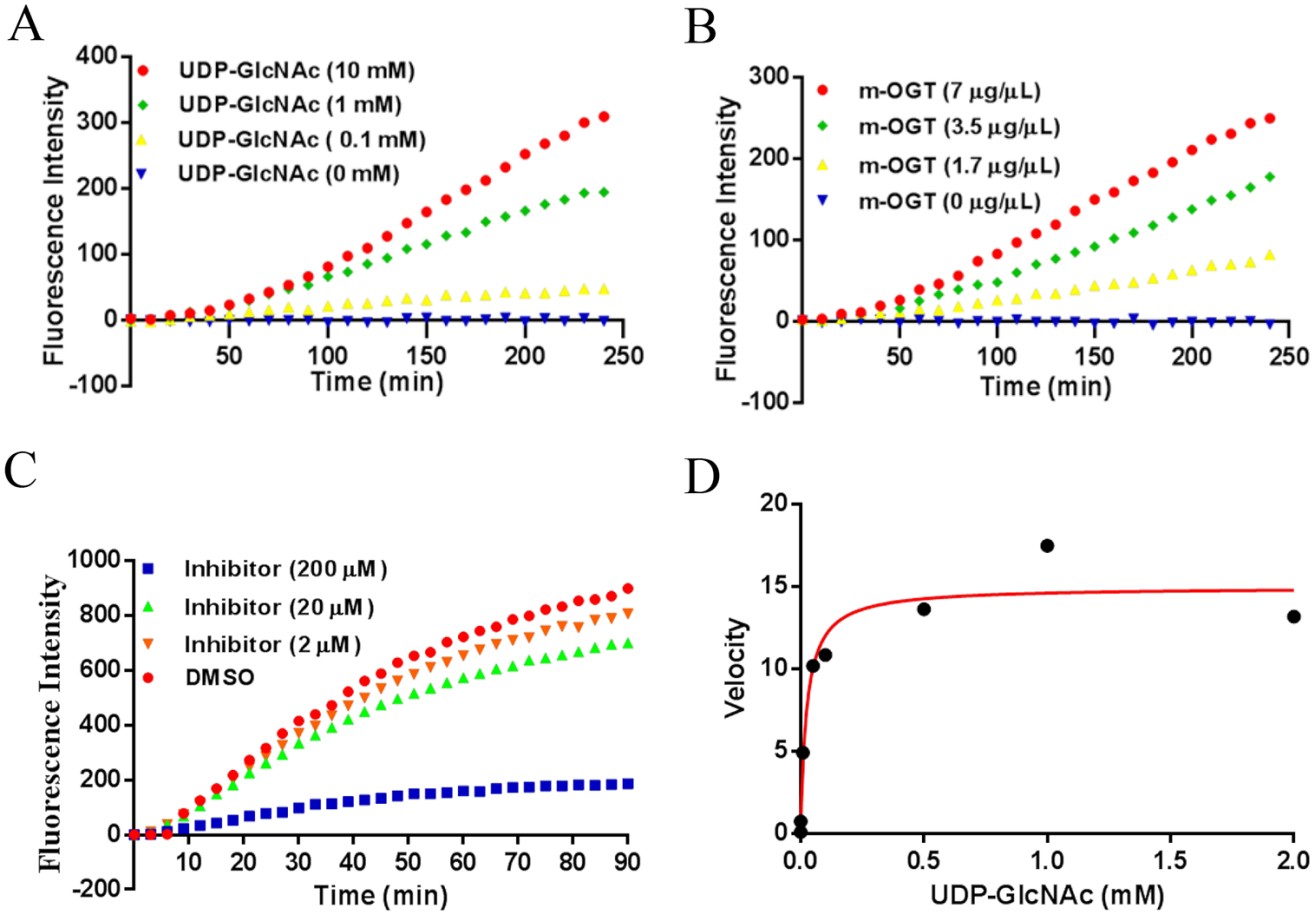
Validation of RBL-2_410–422 O-GlcNAcylation. A, O-GlcNAcylation of RBL-2_410–422 peptide by bacterial lysate containing m-OGT (7 μg/μL) with increasing concentration of UDP-GlcNAc (0–10 mM). B, O-GlcNAcylation of RBL-2_410–422 peptide dependency of increasing total protein concentrations of bacterial lysate containing m-OGT (0, 1.7, 3.5, 7 μg/μL) at 1 mM UDP-GlcNAc. C, O-GlcNAcylation of RBL-2_410–422 peptide by purified m-OGT (0.2 μg/μL) with 1 mM UDP-GlcNAc was inhibited by a known OGT inhibitor (ST045849, 0–200 μM). D, Km value for UDP-GlcNAc was determined with fixed saturating concentration of RBL-2_410–422 peptide, purified m-OGT (0.2 μg/μL) and varying concentration of UDP-GlcNAc (0–2 mM). The Km value derived from the fit to Michaelis-Menten model is 24 μM.

Interestingly, the best hit in our screening assay is the peptide RBL-2_420–422 derived from the protein Retinoblastoma-Like 2 protein, which plays a pivotal role in the regulation of the cell cycle and may function as a tumor suppressor [29, 30]. Its interesting biology motivated us to further focus on the validation of RBL-2 O-GlcNAcylation in the following work. Kinetic signals of the GlcNAcylation were collected to fully understand the O-GlcNAcylation of RBL-2_420–422. As expected, we found that rates O-GlcNAcylation of RBL-2_420–422 increased upon the addition of increasing concentrations of the donor UDP-GlcNAc (Fig 3A). Consistent with this, O-GlcNAcylation of RBL-2_420–422 increased with increasing amounts of bacterial lysate containing recombinant m-OGT (Fig 3B). Next, we tested whether this activity can be suppressed using a known OGT inhibitor (ST045849, S1 Fig) with a reported IC_50_ value around 50 μM [31], and 80% inhibition of RBL-2_420–422 O-GlcNAcylation was observed at 200 μM of ST045849 (Fig 3C). Furthermore, the Km value of the UDP-GlcNAc donor involved in the RBL-2_420–422 O-GlcNAcylation was obtained by incubation of purified m-OGT with varying concentrations of UDP-GlcNAc ranging from 0 to 2 mM on the microarray and this reaction showed Michaelis-Menten behavior with a Km value of 24 μM. Taken together, these data indicated that the protein RBL-2, from which the peptide studied was derived, is a possible substrate of OGT.

### Ser 420 is one of the O-GlcNAc sites in RBL-2

**Fig 4:**
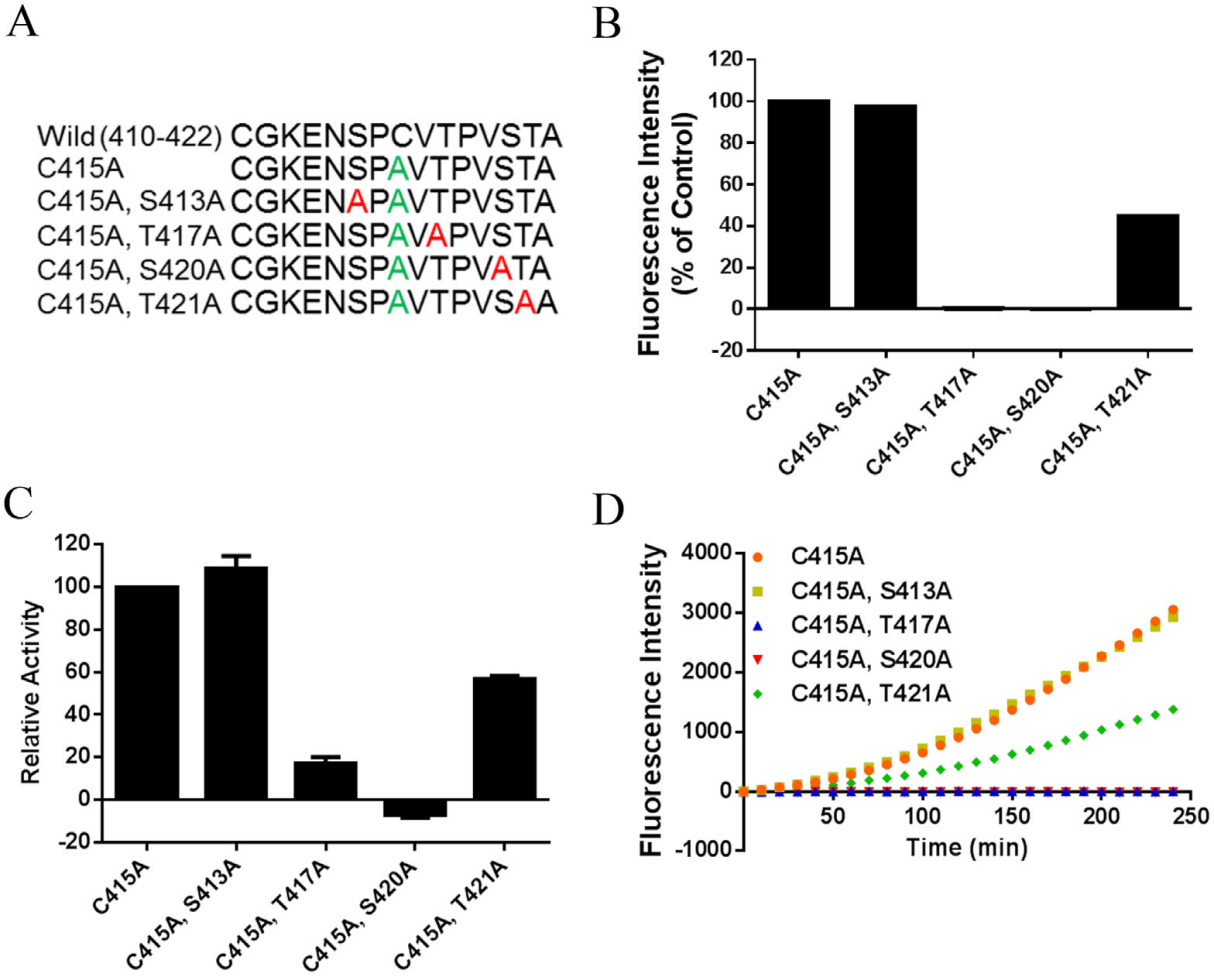
S420A is a possible O-GlcNAc site in the RBL-2 protein. A, peptide mutant used for an Ala scan. For immobilization purposes, peptides were prepared with an extra CG at the N-terminus and Cys 415 was replaced by Ala. B, OGT activity against RBL-2_410–422 peptide mutants was determined using peptide microarray analysis with 0.2 μg/μL purified m-OGT and 1 mM UDP-GlcNAc. C, UDP-Glo assay was used to measure O-GlcNAcylation of RBL-2_410–422 peptide mutants as well. D, kinetic signals from the same microarray experiment of panel B are shown.

Interestingly, the amino acids around Ser 420 (-2 Pro, -1 Val, +1 Thr, and +2 Ala) in this peptide closely matched the previously described amino acids preference of OGT as recently determined[16, 17, 32], suggesting that Ser 420 might be the most likely O-GlcNAc site on this peptide which contains 4 possible sites in total. Also mass spectrometry results indicated that Ser 420 was O-GlcNAcylated in this peptide as recently described [25]. To further confirm this and investigate the role of this and surrounding amino acid residues in our activity based assay, we resynthesized the RBL-2 peptide present on the microarray and performed an Ala scan, thus replacing of all the possible O-GlcNAc sites (Ser and Thr) in RBL-2_420–422 (Fig 4A). To immobilize the peptides on the chip, all the peptides were prepared with an extra CG at the NH2 terminal to allow immobilization on the maleimide-based chip surface. Cys 415 was replaced by an Ala to avoid cyclisation by disulfide bond formation or dual modes of immobilization (S2 Fig). As shown in Fig 4B, substitution of Ser 420 and Thr 417 by Ala resulted in a complete loss of OGT activity against RBL-2_410–422. The mutants T421A decreased the O-GlcNAcylation of RBL-2_420–422 peptide by 40% compared to the control (wild type with C415A). However, mutant S413A did not show any effect on the O-GlcNAcylation of RBL-2_420–422. In addition, OGT activity against these peptides was further validated using the UDP-Glo assay, which measures the amount of UDP produced when GlcNAc is transferred from UDP-GlcNAc to the sugar accepting peptide. Notably, O-GlcNAcylation activities of S413A, S420A, and T421A measurements in solution were consistent with the microarray results, except that T417A still showed 20% O-GlcNAcylation in this assay. This might be because the UDP assay is more sensitive than the antibody-based detection.

In conclusion, these microarray results are consistent with the mass spectrometry and X-ray observations[25] and confirmed that Ser 420 is one of the possible O-GlcNAc sites of RBL-2. Our findings further indicated that T417 and T421 make important contributions to OGT substrate recognition.

### RBL-2 S420A peptide showed inhibitory effect against OGT

**Fig 5:**
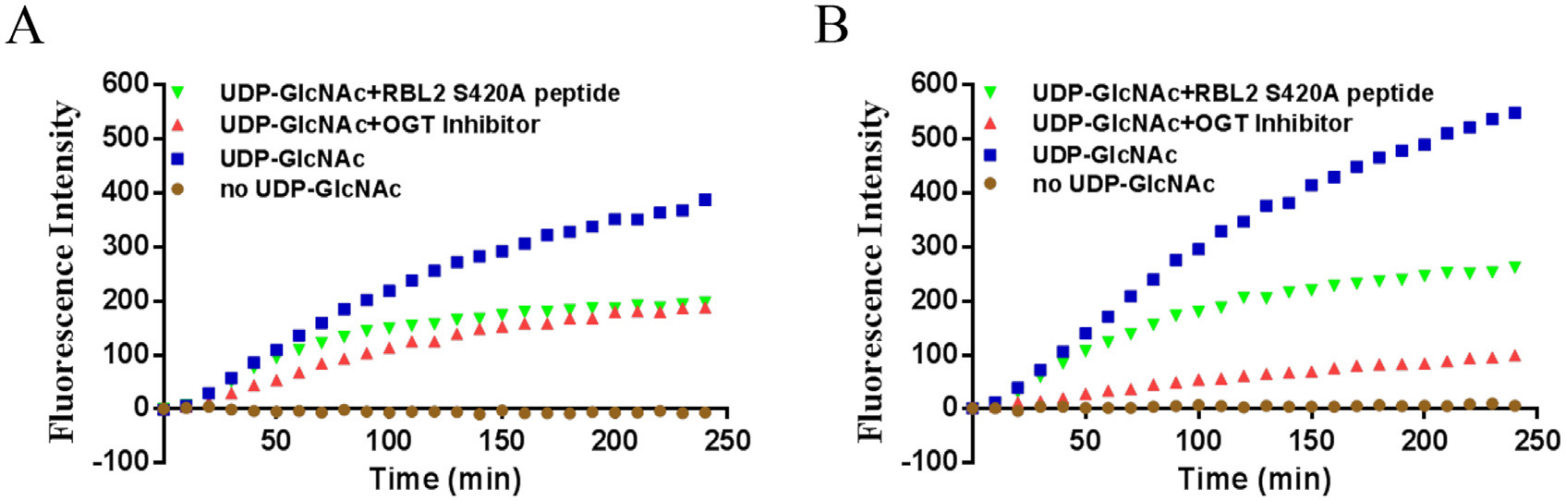
The RBL-2_S420A 410–422 peptide inhibited OGT activity. The inhibitory effect of RBL-2_S420A 410–422 peptide on OGT activity was determined on the nuclear receptor interaction peptide microarray. The reaction was performed by incubating a mixture of purified m-OGT (0.2 μg/μL) and UDP-GlcNAc (0.5 mM) with or without the S420A RBL2 peptide (0.5 mM). The known OGT inhibitor (ST045849) and a no UDP-GlcNAc reaction were used for positive and negative control, respectively. O-GlcNAcylation of NCOA6_1479–1501 peptide (A) and WIPI_1313–318 peptide (B) are shown for the inhibitory effect of RBL-2_S420A 410–422 peptide on OGT activity.

We then used the identified substrate sequence as a starting point towards the discovery of novel selective OGT inhibitors[33]. Thus was further motivated by the fact that it has been described that a RBL-2 derived small peptide showed in vivo antitumor activity by disruption of CDKs kinase activity[34]. In addition, in very recent work, a crystal structure study has demonstrated that the RBL-2 derived peptide can bind to the OGT catalytic sites by forming many specific hydrogen bonds and Waals interactions[25]. Given that the RBL-2_420–422 peptide can be highly O-GlcNAcylated and S420A mutation resulted in the completely abolishment of OGT activity against RBL-2_420–422 peptide, we hypothesized that S420A mutation peptide might still bind to the active site of OGT and competitively prevent substrate binding to the catalytic domain. To test it, we determined its inhibitory effect on the O-GlcNAcylation of two OGT substrates identified in our work and a known OGT inhibitor was used as a positive control. We found that O-GlcNAcylation of NCOA6 and WIPI 1 was inhibited by 50% in the presence of 500 μM of the S420A peptide (Fig 5). Thus, these data indicated that RBL-2_ S420A 410–422 still binds to the OGT active site and therefore inhibited OGT activity.

## Discussion

Protein microarrays have been demonstrated as highly efficient analytical tools for protein modification studies, especially phosphorylation[35]. More recently, two groups described the use of high density human protein microarrays to discover OGT substrates and OGT interactors, respectively [21, 36]. The dynamic PamChip peptide microarray has provided a very powerful technique for kinase activity profiling, and here we first describe its utility in the study of O-GlcNAcylation activity. With this approach, several peptides derived from kinase substrates and nuclear receptor binding co-regulators were identified in this current work, and our results further demonstrated that this peptide microarray is a very useful tool for studying the O-GlcNAcylation activity.

Recently, the amino acid preference of OGT around the O-GlcNAc site has been reported several times using different approaches [16, 17, 25, 32]. Coincidentally, we found that four amino acids around Ser 420 in RBL-2_420–422 peptide completely matched with the best motif preferred by OGT. Finally we and others demonstrated that RBL-2 Ser 420 is one of the O-GlcNAcylated sites. Thus it partially explained the OGT specificity and it might be helpful for the specificity based identification of OGT substrates. We further compared the described sequons[16, 17, 25, 32] with all peptides displayed on the microarrays by a FuzzPro search[37]. It was found that three of the kinase substrate peptides (GYS2_1_13, RBL-2_410_422 and RBL-2_655_667) and four of the nuclear receptor interaction peptides (DHX30_241_262, NCOA1_620_643, NCOA6_1479_1501 and PELP1_20_42) match the O-GlcNAc sequons. Interestingly, only two of these sequon-matching peptides (NCOA1_620_643 and PELP1_20_42) were not identified in our microarray assay and at the same time some of the microarray identified hits did not match with the sequons. Clearly the current sequons are not complete. Nevertheless the identification of five sequon-matching-substrate sequences further validated our peptide microarray screening method. Furthermore, while previous work indicates a requirement of an extended conformation of the substrate[25] the nuclear receptor co-activator derived peptides are known to have a helical motif[38, 39].

Abnormal cell cycle control is one of the characteristics in cancer cells. The expression of RBL-2 has been discovered both in normal human tissues and cancer [40]. RBL-2 and other two retinoblastoma family members (pRb and p107) play fundamental roles in the negative regulation of the cell cycle and have been demonstrated as tumor suppressors[41]. pRb and p107 were shown to be O-GlcNAcylated both in vitro and in vivo[42]. We and others showed that RBL-2_420–422 can be modified by O-GlcNAc using a peptide array. These studies indicated that not only phosphorylation but also the O-GlcNAcylation are likely involved in the regulation of RBL-2 in cell cycle control. However, it remains to be determined if O-GlcNAcylation of RBL-2 happens in vivo and if it affects cell cycle progression.

The binding motif involved in substrate-enzyme interactions and protein-protein interaction can be used to develop selectively inhibitors. Indeed, an OGT inhibitor based on RBL-2 spacer domain showed promising antitumor effect [34]. In the present work, we found RBL-2_S420A 420–422 peptide showed significant inhibition of OGT activity. Fortunately, the crystal structure of OGT in complex with RBL-2 peptide was reported recently and it was indicated that hydrogen bonds and a hydrophobic environment around the O-GlcNAc site are responsible for the binding of the RBL-2 peptide to OGT. These findings are increasing the possibility for the development of potent selective OGT inhibitor based on RBL-2_S420A 420–422 peptide.

In summary, we herein described the application of a peptide microarray approach in the discovery of OGT substrates and the study of its enzymatic properties. Using this array, RBL-2 was discovered as an OGT substrate and its O-GlcNAc site was identified. In our experiments a high substrate specificity was observed, which may at least in part explain the existence of few OGT’s. Our data are indicative of the usefulness of the peptide microarray approach for the identification of OGT activity and will likely prove valuable in diagnosis of GlcNAcylation-linked disease and its interplay with other post-translational modifications.

## Supporting Information

S1 Appendix Sequences of peptides displayed on kinase peptide microarray. (XLSX)

S2 Appendix Sequences of peptides displayed on nuclear hormone receptor interaction peptide microarray. (XLSX)

S1 Fig The known OGT inhibitor (ST045849) used in this study. (TIF)

S2 Fig C415A showed modest effect on O-GlcNAcylation of RBL-2-410-422. O-GlcNAcylation of wild type RBL-2-410-422 and C415A peptide was determined using both the UDP assay(A) and the microarray assay (B) with 1 mM UDP-GlcNAc and 0.2 μg/μL purified m-OGT. Compared with wild type, the C415A peptide showed modest increase in OGT activity in the UDP-assay. However, in microarray assay the wild type peptide showed dramatic decrease of activity comparing with C415A peptide.
(TIF)
