# Supplementary figures and images for: Activity Based High-Throughput Screening for Novel O-GlcNAc Transferase Substrates Using a Dynamic Peptide Microarray
Source: PLoS One. 2016 Mar 9;11(3):e0151085. doi: 10.1371/journal.pone.0151085 (PMC4784888; doi:10.1371/journal.pone.0151085)

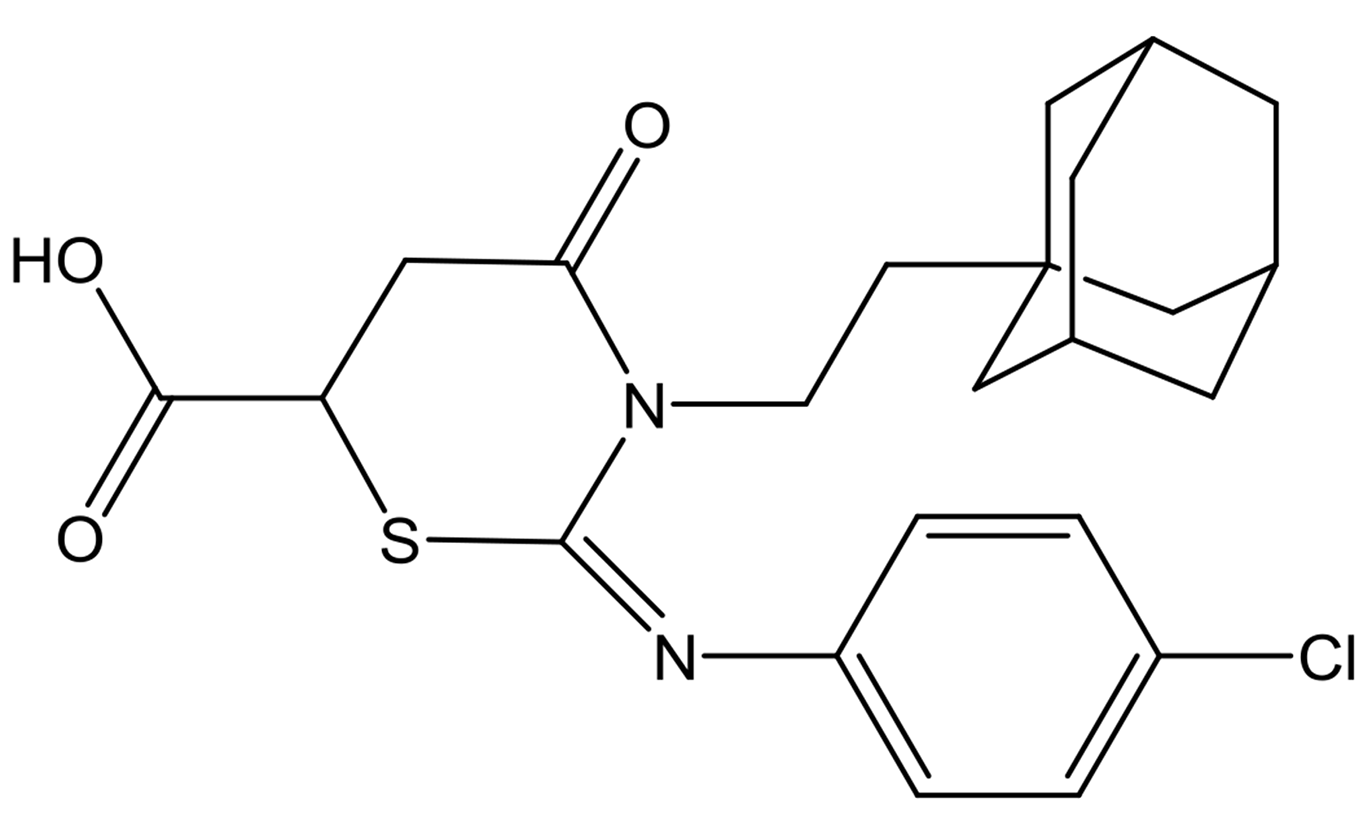

Supplement: S1 Fig — (TIF) [file pone.0151085.s003.tif]

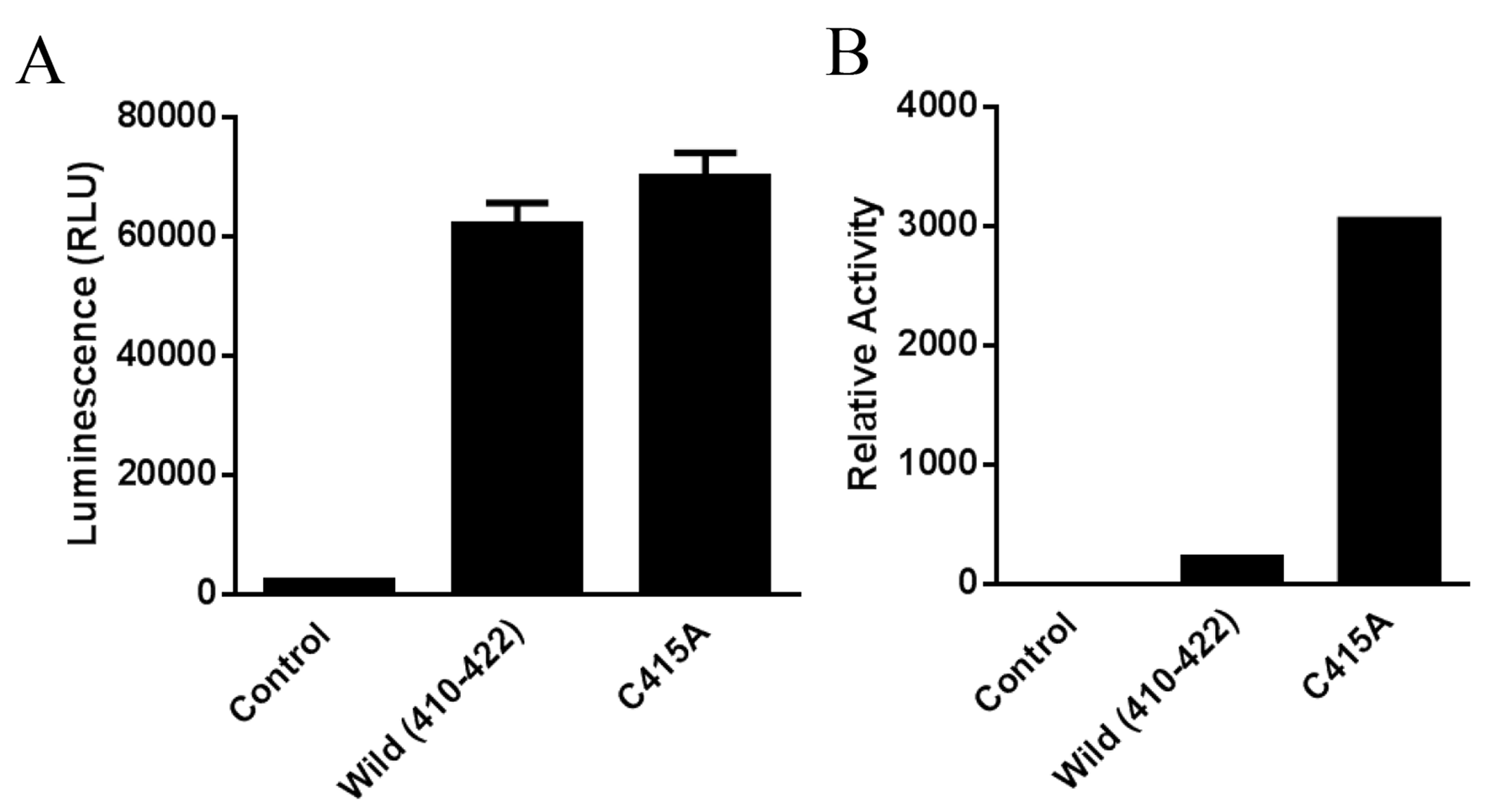

Supplement: S2 Fig — O-GlcNAcylation of wild type RBL-2-410-422 and C415A peptide was determined using both the UDP assay(A) and the microarray assay (B) with 1 mM UDP-GlcNAc and 0.2 μg/μL purified m-OGT. Compared with wild type, the C415A peptide showed modest increase in OGT activity in the UDP-assay. However, in microarray assay the wild type peptide showed dramatic decrease of activity comparing with C415A peptide. (TIF) [file pone.0151085.s004.tif]
